# Supplementary material for: In Silico RNAseq and Biochemical Analyses of Glucose-6-Phosphate Dehydrogenase (G6PDH) from Sweet Pepper Fruits: Involvement of Nitric Oxide (NO) in Ripening and Modulation
Source: Plants (Basel). 2023 Sep 27;12(19):3408. doi: 10.3390/plants12193408 (PMC10574341; doi:10.3390/plants12193408)
Supplement: Supplementary file 1 [file plants-12-03408-s001.zip › plants-2509341-supplementary.pdf]

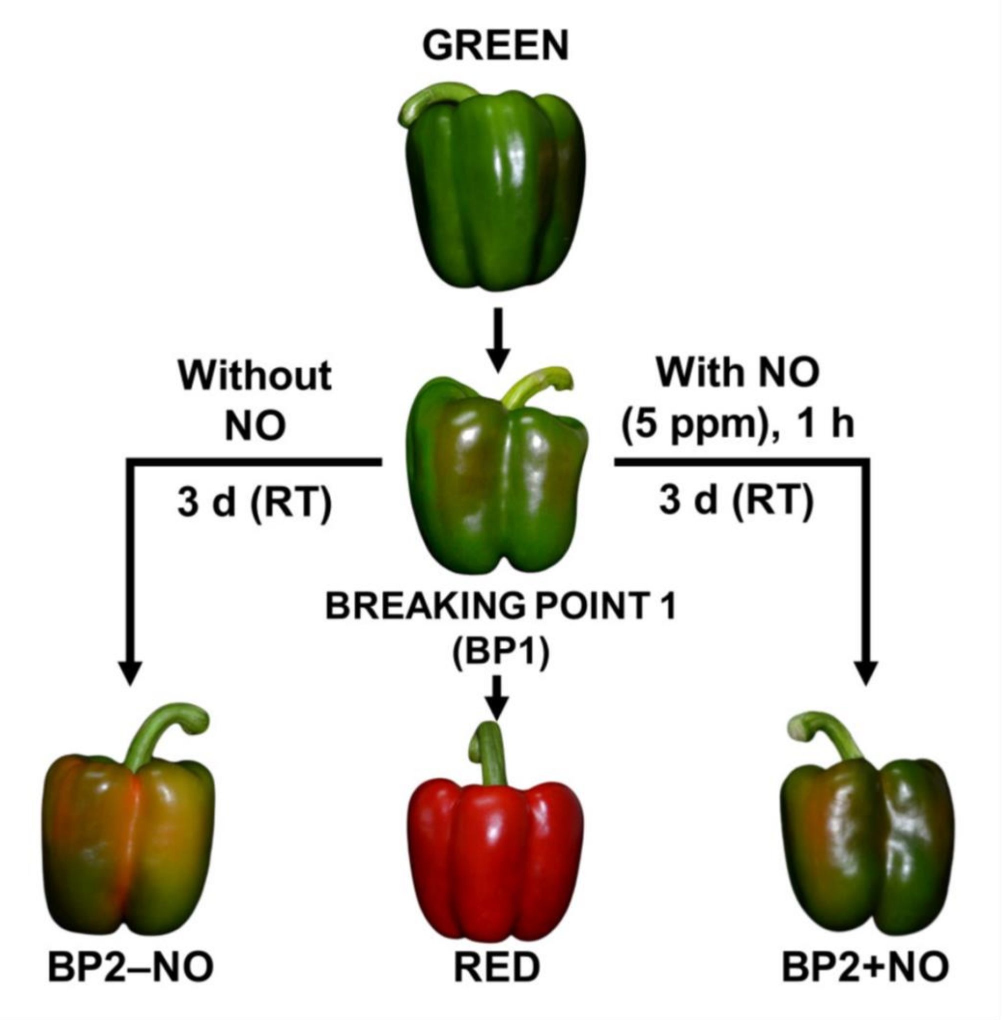

Supplementary Figure S1. Illustrative picture showing the experimental design used in this study with the representative phenotype of sweet pepper (*Capsicum annuum* L.) fruits at different stages and treatments: immature green, breaking point 1 (BP1), breaking point 2 without NO treatment (BP2 – NO), breaking point 2 with NO treatment (BP2 + NO), and ripe red. Fruits were subjected to a NO-enriched atmosphere (5 ppm) in a hermetic box for 1 h and were then stored at room temperature (RT) for 3 days. Reproduced with permission from González-Gordo et al. (2022b) [46].

Supplementary Table S1. List of the 53 different isozymes of G6PDH belonging to 15 different plant species used in this work for phylogenetic analysis.

Plant species	G6PDH isozyme	Protein ID	Reference
Pepper<br>(*Capsicum annuum)*	CaG6PDH1<br>CaG6PDH2<br>CaG6PDH3<br>CaG6PDH4	XP_016561682.1<br>XP_016570698.1<br>XP_016580465.1<br>XP_016573493.1	The present study
Arabidopsis thaliana	AtG6PDH1<br>AtG6PDH2<br>AtG6PDH3<br>AtG6PDH4<br>AtG6PDH5<br>AtG6PDH6	Q43727.2<br>Q9FY99.2<br>Q8L743.2<br>Q93ZW0.1<br>Q9LK23.1<br>Q9FJI5.1	Wendt et al., 1999; Wakao and Benning, 2004)
Alfalfa<br>(*Medicago sativa*)	MsG6PDH1	AAB41552.1	(Wendt et al., 1999)
Tobacco<br>(*Nicotiana tabacum*)	NtG6PDH1<br>NtG6PDH2<br>NtG6PDH3	CAA04994.1<br>AAF87216.1<br>CAA04992.1	(Wendt et al., 1999)
Rice<br>(*Oryza sativa*)	OsG6PDH1<br>OsG6PDH2<br>OsG6PDH3	AAS07054.1<br>BAC84352.1<br>CAC09489.2	(Wendt et al., 1999)
Parsley (*Petroselinum crispum*)	PcG6PDH1<br>PcG6PDH2<br>PcG6PDH3	AAB69318.1<br>AAB69319.1<br>AAB69317.1	(Wendt et al., 1999)
Potato (*Solanum tuberosum*)	StG6PDH1<br>StG6PDH2<br>StG6PDH3	CAA52442.1<br>CAA58775.1<br>CAB52708.1	(Wendt et al., 1999)
Spinach (*Spinacia oleracea*)	SoG6PDH1	CAA03939.1	(Wendt et al., 1999)
Wheat (*Triticum aestivum*)	TaG6PDH1	BAA97663.1	(Wendt et al., 1999)
Rubber tree (*Hevea brasiliensis*)	HbG6PDH1<br>HbG6PDH2<br>HbG6PDH3<br>HbG6PDH4	AIE47266.1<br>AIE47267.1<br>AIE47268.1<br>AIE47269.1	(Long et al., 2016)
Black cottonwood<br>(*Populus trichocarpa*)	PtG6PDH1<br>PtG6PDH2<br>PtG6PDH3<br>PtG6PDH4	EEE79649.2<br>ERP53365.1<br>EEE94856.1<br>EEF03929.1	(Long et al., 2016)
Castor oil<br>(*Ricinus communis*)	RcG6PDH1<br>RcG6PDH2<br>RcG6PDH3	EEF47431.1<br>EEF32168.1<br>EEF50009.1	(Long et al., 2016)
Maize<br>(*Zea mays*)	ZmG6PDH1<br>ZmG6PDH2<br>ZmG6PDH3<br>ZmG6PDH4	AFW57831.1<br>DAA45780.1<br>NP_001348206.1<br>ACG39996.1	(Long et al., 2016)
Tomato<br>(*Solanum lycopersicum*)	SlG6PDH1<br>SlG6PDH2<br>SlG6PDH3<br>SlG6PDH4	XP_004230338.1<br>XP_004231802.1<br>XP_004239245.1<br>XP_004243567.1	Tomato proteome

Soybean<br>(*Glycine max*)	GmG6PDH1<br>GmG6PDH2<br>GmG6PDH3<br>GmG6PDH4<br>GmG6PDH5<br>GmG6PDH6<br>GmG6PDH7<br>GmG6PDH8<br>GmG6PDH9	XP_003520786.1<br>NP_001241264.1<br>XP_003533032.1<br>XP_003547679.1<br>XP_003518668.1<br>XP_003553875.1<br>XP_003552660.1<br>XP_014633122.1<br>XP_003554604.1	(Zhao et al., 2020)

References

Long, X., He, B., Fang, Y., & Tang, C. (2016). Identification and characterization of the glucose-6-phosphate dehydrogenase gene family in the para rubber tree, *Hevea brasiliensis*. *Frontiers in Plant Science*, *7,* 215. https://doi.org/10.3389/fpls.2016.00215

Wakao, S., & Benning, C. (2004). Genome-wide analysis of glucose-6-phosphate dehydrogenases in Arabidopsis. *The Plant Journal*, *41*, 243–256. https://doi.org/10.1111/j.1365-313x.2004.02293.x

Wendt, U. K., Hauschild, R., Lange, C., Pietersma, M., Wenderoth, I., & von Schaewen, A. (1999). *Plant Molecular Biology*, *40*, 487–494. https://doi.org/10.1023/a:1006257230779

Zhao, Y., Cui, Y., Huang, S., Yu, J., Wang, X., Xin, D., Li, X., Liu, Y., Dai, Y., Qi, Z., & Chen, Q. (2020). Genome-wide analysis of the glucose-6-phosphate dehydrogenase family in soybean and functional identification of GMG6PDH2 involvement in salt stress. *Frontiers in Plant Science*, *11*, 214. https://doi.org/10.3389/fpls.2020.00214
